# Emerging trends and research hotspots of non-invasive brain stimulation for stroke: a bibliometric and visualization study

**DOI:** 10.3389/fneur.2025.1540405

**Published:** 2025-05-20

**Authors:** Zhengyu Li, Xi Zhao, Siyu Xie, Wenying Shi, Wei Zhang

**Affiliations:** ^1^School of First Clinical Medical, Hunan University of Chinese Medicine, Changsha, Hunan, China; ^2^School of Acupuncture-Moxibustion and Tuina, Hunan University of Chinese Medicine, Changsha, Hunan, China; ^3^Acupuncture and Rehabilitation Center, The First Hospital of Hunan University of Chinese Medicine, Changsha, Hunan, China

**Keywords:** stroke, non-invasive brain stimulation, rehabilitation, bibliometric analysis, visualization

## Abstract

**Objectives:**

With the advent of an aging population society, the morbidity and mortality rates of stroke are on the rise. Most surviving patients are often accompanied by a series of sequelae, which seriously affect patients’ social function and physical and mental health. The application of non-invasive brain stimulation (NIBS) in neurorehabilitation has attracted widespread attention. This study aims to explore the key theme and future direction of the research in this field.

**Methods:**

Articles and reviews related to NIBS for stroke from January 1985 to September 2024 were identified from the Web of Science Core Collection database. The CiteSpace, VOSviewer software, and Charticulator website were used to visualize and analyze the publications, countries, institutions, authors, journals, keywords, cited references, subject categories, and funding agencies from various angles.

**Results:**

A total of 4,453 papers were included in this study, with the United States publishing the most, followed by China. The most outstanding author was Fregni F from Harvard Medical School. Frontiers in Neurology had the highest number of publications. Plasticity and excitability represent two particularly major themes, and connectivity is the keyword of the research frontier in recent years.

**Conclusion:**

NIBS shows considerable potential and broad development space in stroke rehabilitation. This study analyses the research hotspots and emerging trends in this field, thereby providing a framework for deeper research and contributing to the vigorous development of NIBS for stroke.

## Highlights

- Most patients of stroke are often accompanied by a series of sequelae, which seriously affect patients' social function and physical and mental health.
- NIBS has significant therapeutic value and potential in the field of stroke rehabilitation.
- The research in this field is gradually shifting from clinical medicine to fundamental disciplines and social sciences.
- Functional connectivity of brain networks may be the future direction of mechanism research.

## 1 Introduction

Stroke is an acute cerebrovascular disease that causes damage to brain tissue, primarily due to the interruption of blood circulation within the brain. There are two main types of stroke: ischemic stroke (IS) and hemorrhagic stroke (HS) (1). The 2019 Global Burden of Disease (GBD) database indicates that stroke still has high rates of disability and remains the second leading cause of death globally, second only to heart disease (2). In recent years, the incidence of stroke has shown an alarming increase and tends to be younger due to unhealthy lifestyles and food safety issues, making it one of the major challenges to global health (3). In China, the prevalence of stroke in 2021 has increased by 104.26% since 1990, stroke is still the leading cause of health loss (4). The long-term prognosis of stroke patients is dependent on several factors, including the extent of brain damage and the duration of treatment. Individuals with severe brain injuries may suffer long-term disability or even mortality (5). With the advancement of medical care, the survival rate of stroke patients has been greatly improved. However, many patients suffer from severe neurological dysfunction due to missing the optimal time window. This not only affects the individual but also places a considerable economic burden on families and society at large (6). Therefore, it is imperative to improve post-stroke dysfunction and enhance patients’ ability to live independently and quality of life.

With the development of neurological disciplines and the advancement of technical means, brain stimulation has received more and more attention in the field of neurorehabilitation, and non-invasive brain stimulation (NIBS) is one of them (7). NIBS is a technique for modulating brain function that involves applying energy from physical factors, such as electricity, magnetism, or ultrasound, to the brain without damaging the scalp and skull. This approach is used to alter specific neural activities and behaviors. Unlike physical therapy, occupational therapy, and other forms of stroke rehabilitation, NIBS can directly modulate neural activity in specific brain regions and networks, thereby promoting neuronal plasticity and reconstruction of neural circuits (8, 9). In recent years, NIBS has been widely used in stroke rehabilitation due to its advantages of convenience, feasibility, non-invasiveness, cost-effectiveness, and efficacy. Numerous studies have confirmed that NIBS can significantly improve dysfunctions after stroke, including dysphagia, aphasia, dyskinesia, cognitive impairment, and depression. Additionally, NIBS has been shown to regulate the content and distribution of neurotransmitters in the brain, enhance the microenvironment of neurons, and influence the remodeling of synaptic function (10–14). NIBS has significant therapeutic value and potential in the field of stroke rehabilitation and has become one of the research hotspots in related academic fields both domestically and internationally.

Bibliometrics is a discipline that takes literature as the research object and explores the internal structural characteristics and patterns within a specific field through mathematical, statistical, and other measurement methods. This discipline plays a crucial role in the evaluation of scientific research and the planning of disciplinary development, characterized by its highly quantitative nature and strong practical applicability. At present, some researchers have conducted bibliometric analysis on transcranial magnetic stimulation (TMS) or transcranial direct current stimulation (tDCS) for stroke (15–19). The scope of this study is much broader, involving all NIBS techniques for stroke. This approach seeks to provide a systematic, objective, and comprehensive understanding of the current status, research hotspots, and emerging trends in the application of NIBS within the field of stroke rehabilitation. Ultimately, the findings aim to offer research directions for further inquiry and practice by rehabilitation clinicians and researchers.

## 2 Materials and methods

### 2.1 Data source and search strategy

The data for this study were sourced from the Web of Science Core Collection (WoSCC), which includes the Science Citation Index Expanded (SCI-Expanded), Social Sciences Citation Index (SSCI), Arts & Humanities Citation Index (AHCI), Conference Proceedings Citation Index-Science (CPCI-S), Conference Proceedings Citation Index-Social Science & Humanities (CPCI-SSH), Emerging Sources Citation Index (ESCI), Current Chemical Reactions (CCR-Expanded), and Index Chemicus (IC). Web of Science contains a wide range of international academic journals and stands as one of the most comprehensive and authoritative database platforms for obtaining global academic information, which highly represents academic development in a particular field (20). To avoid potential bias caused by database updates, literature retrieval and data extraction were performed on the same day.

**Figure 1:**
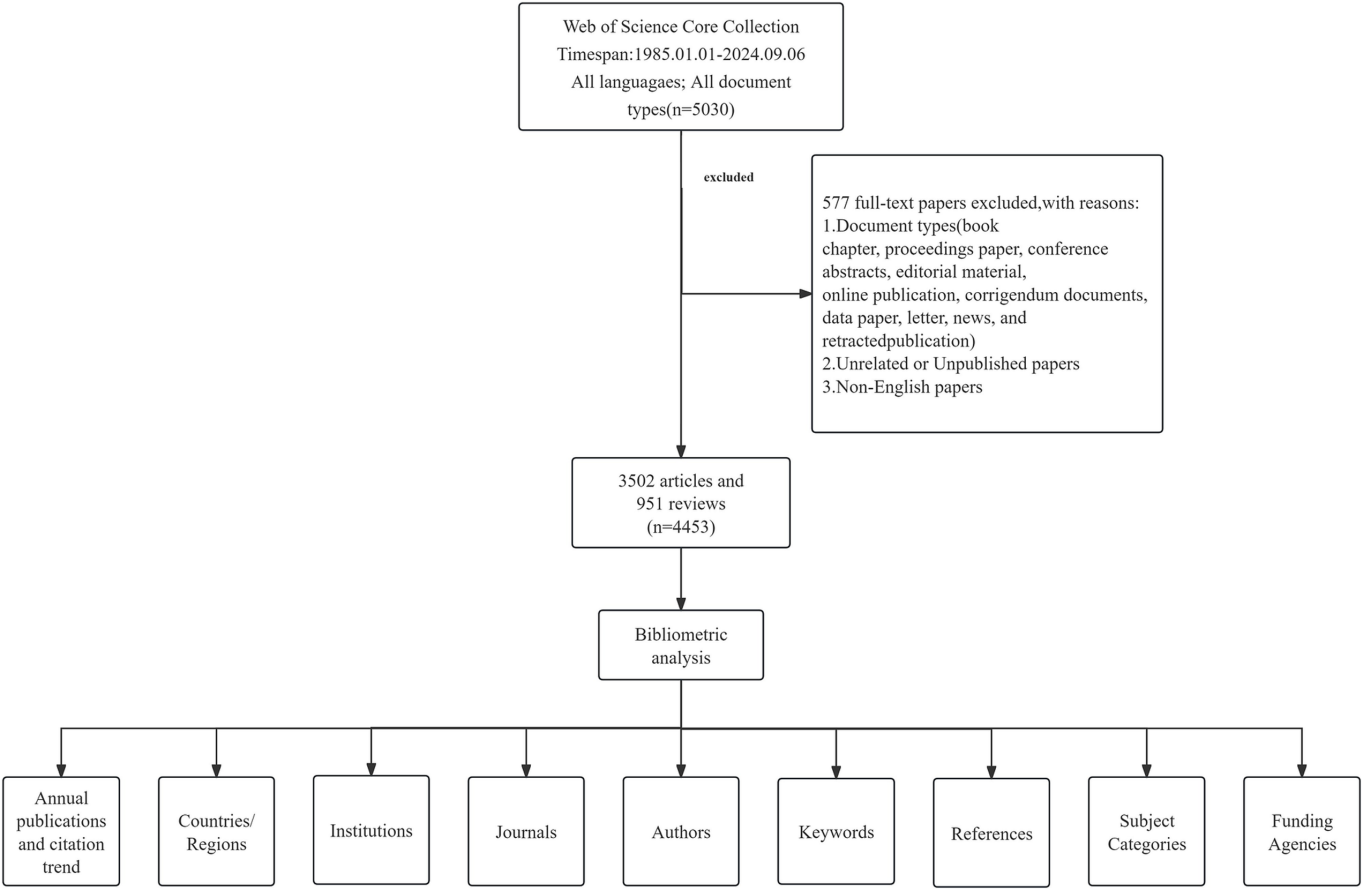
Flowchart of the process for the study.

To ensure a comprehensive search, subject terms were derived from the Medical Subject Headings (MeSH) database^1^, and a combination of these MeSH terms and related terms was employed, incorporating truncations (*) to increase the search rate. The search strategy is detailed in Supplementary material 1. The search time range was from January 1, 1985, to September 6, 2024. The search document type was set to “Article” and “Review,” and the document language was set to English. To ensure data accuracy, two researchers manually reviewed publications following predefined criteria. Any discrepancies encountered would be resolved by a third professional (Figure 1).

### 2.2 Exclusion criteria

(1) Literature related to book chapters, proceeding papers, meeting abstracts, editorial materials, letters, retracted publications, notes, news items, and corrections; (2) Irrelevant, duplicated, or unpublished literature; and (3) Literature not in English.

### 2.3 Bibliometric analysis

Literature that met the inclusion and exclusion criteria was exported to plain text named “download_xxx.txt” with “full record and cited references.” The exported documents were subsequently imported into the Charticulator online website, VOSviewer 1.6.20, and CiteSpace 6.3.R1 software for the construction of knowledge graphs and statistical analyses. The Charticulator online website, VOSviewer, and CiteSpace software are powerful tools for the analysis of scientific research literature, each with its unique features and advantages.

The Charticulator^2^ is a powerful and free online visualization platform developed by Microsoft Research. This website was used to conduct the collaborative analysis of countries (21).

VOSviewer is a free, Java-based bibliometric software tool that facilitates the construction and visualization of scientific literature networks. It generates detailed and comprehensible network maps based on various relationships (22). In this study, VOSviewer software was used for co-occurrence analysis of countries, institutions, authors, journals, cited references, and keywords. The count mode is full count, the minimum citation frequency is set appropriately, and the rest of the parameters are the default settings of the software. The node represents an element, node size indicates the number of literature, the connecting lines between nodes illustrate the collaborative relationships, and its thickness reflects the degree of closeness and extent of these connections. A thicker line signifies a closer relationship, while a greater number of lines indicates more extensive cooperation with other nodes, positioning it in a relatively core role and the higher its influence. Additionally, the color of the nodes represents the clusters formed by their cooperation.

CiteSpace is another free Java-based bibliometric software developed by Chen and Song, primarily designed to assist researchers in identifying and analyzing research hotspots and emerging trends within specific research areas (23). In this study, CiteSpace was used to generate the burst maps of countries, institutions, authors, and references, as well as the clustering and burst maps of keywords. Parameter settings: the period from January 1990 to September 2024, time slice is 1 year, TopN = 50, TopN% = 10.0%, the pruning methods are pathfinder and pruning sliced networks, while the rest are maintained at the system default settings.

### 2.4 Research ethics

The data sources for this study were obtained from public databases and no human subjects were involved in the study, therefore ethical approval was not required.

## 3 Results

### 3.1 Annual publications and citation trend

**Figure 2:**
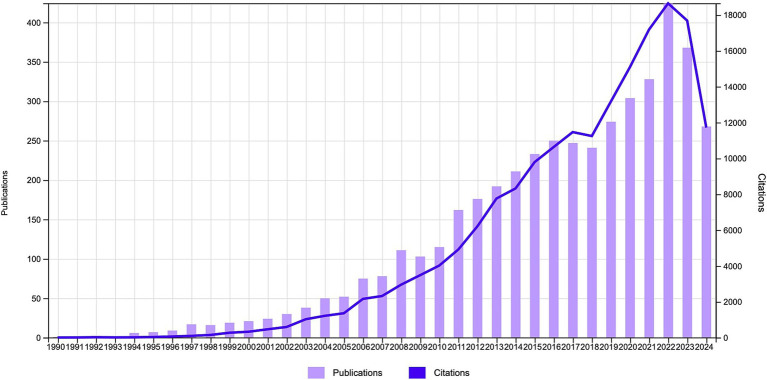
Annual publication outputs and citation trends regarding NIBS for stroke.

From 1985 to 2024, a total of 5,030 relevant publications were published in the WoSCC database concerning NIBS for stroke. After eliminating irrelevant literature types, duplicates, and non-English literature, 3,502 articles and 951 reviews were extracted. Figure 2 illustrates the annual publications and citation trend of the literature related to NIBS for stroke. Over the past 30 years, the annual publications in this field showed an upward trend, while the peak occurred in 2022. This growth can be roughly categorized into three stages: The first stage, from 1990 to 2010, was characterized by a slow annual growth rate, with the number of publications and citations remaining below 150 per year. The second stage is from 2011 to 2018, with a flat and gradual growth in related studies. The third stage, from 2019 to 2024, demonstrated a rapid growth trend and reached a peak in 2022, which indicated that the NIBS has been widely used in stroke rehabilitation with the development of neurological discipline and the advancement of technical means. The decline in the number of publications and citations in 2024 may be attributed to the inclusion of only 9 months of literature, but the overall trend remained stable and at a high level, reflecting the continuity and significance of NIBS in stroke rehabilitation. All publications were cited a total of 184,478 times, with an average of 41.43 citations per publication and an H-Index of 180.

### 3.2 Analysis of countries/regions

**Figure 3:**
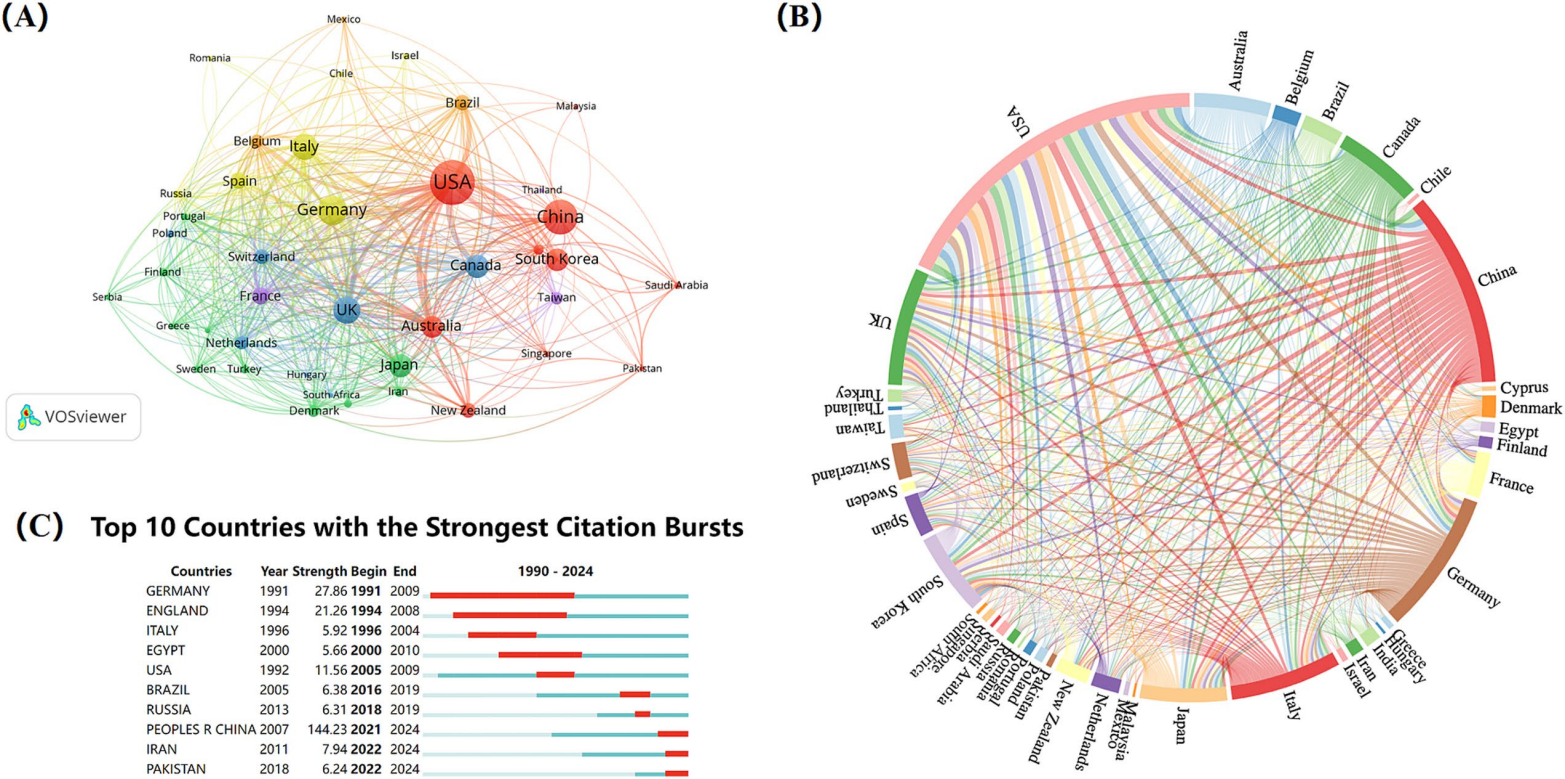
Analysis of countries/regions on NIBS for stroke. **(A)** Co-authorship analysis of countries/regions. The analysis was performed using VOSviewer with the method linlog/modularity, Eight occurrences were included only. **(B)** International collaboration analysis among different countries/regions. **(C)** Top 10 countries with the strongest citation bursts. Light blue indicates that the keyword has not yet appeared. Dark blue indicates that keywords have started to appear, but there have been no significant changes during the period. Red indicates the duration of the strongest citation.

Analyzing the countries/regions of corresponding authors (Figure 3A), a total of 82 countries/regions have contributed to research in this field. The United States, China, Germany, and the United Kingdom emerged as the most active contributors, followed by Italy and Japan. In the map of the country/region cooperation network (Figure 3B), the thickness of the connecting lines is proportional to the degree of cooperation. Thus, thicker lines indicate closer collaborations between countries. Research on NIBS for stroke involved a wide range of countries, with the United States and Germany establishing the most collaborations with other nations, highlighting the importance of international cooperation for comprehensive research in this field. Table 1 illustrates the top 10 countries/regions with the most publications regarding NIBS for stroke. The United States ranked first (1,184, 26.60%), followed by China (697, 15.65%) and Germany (508, 11.41%). Collectively, these three countries accounted for 53.66% of the total publications, underscoring their significant contributions to the field. Additionally, the United States has the highest total citations (67,327) and total link strength (839), while the United Kingdom has the highest average citations (85.03).

**Table 1:** Top 10 countries/regions with most publications regarding NIBS for stroke.

Rank	Country	Documents	Citations	The average cited frequency	Total link strength
1	USA	1,184	67,327	56.86	839
2	China	697	8,573	12.30	159
3	Germany	508	41,617	81.92	532
4	UK	419	35,626	85.03	464
5	Italy	396	26,738	67.52	431
6	Japan	310	11,177	36.05	143
7	Canada	305	12,596	41.30	305
8	South Korea	295	6,600	22.37	89
9	Australia	273	15,880	58.17	369
10	France	162	11,539	71.23	260

Burst terms are keywords that exhibit the highest change rates over a specific period, reflecting historical changes, emerging trends, and future hotspots of research within the field. Among the top ten countries/regions with the strongest citation bursts (Figure 3C), China demonstrated the highest burst strength (144.23), with the burst years primarily concentrated in 2021–2024. This indicates that many Chinese researchers have recently engaged in NIBS techniques for stroke rehabilitation. Germany and the United Kingdom followed closely, with burst strength of 27.86 and 21.26, respectively.

### 3.3 Analysis of institutions

A total of 4,015 institutions published literature on NIBS for stroke. Table 2 lists the top ten relevant institutions with the highest number of publications, half of which are from the United States. Harvard University published the most literature, followed by University College London and the University of Auckland. The top three institutions with the most citations are Harvard University (12,355), University College London (9907) and Neurological Disease and Stroke (8052). It is worth noting that Harvard University is at the top of the list in terms of number of publications, number of citations and Total link strength, which to some extent reflects its high academic impact and capability to produce influential research outcomes.

**Table 2:** Top 10 institutions with most publications regarding NIBS for stroke.

Rank	Institutions	Countries	Documents	Citations	The average cited frequency	Total link strength
1	Harvard University	USA	115	12,355	107.43	223
2	University College London	UK	94	8,052	85.66	143
3	University of Auckland	New Zealand	87	5,103	58.66	113
4	Harvard Medical School	USA	76	3,128	41.16	202
5	Yeungnam University	South Korea	76	1735	22.83	27
6	Johns Hopkins University	USA	74	3,807	51.45	128
7	Northwestern University	USA	69	2,487	36.04	130
8	National Institute of Neurological Disease and Stroke	USA	68	9,907	145.69	161
9	Jikei University	Japan	67	1,352	20.18	49
10	Universidade de São Paulo	Brazil	67	4,391	65.54	144

**Figure 4:**
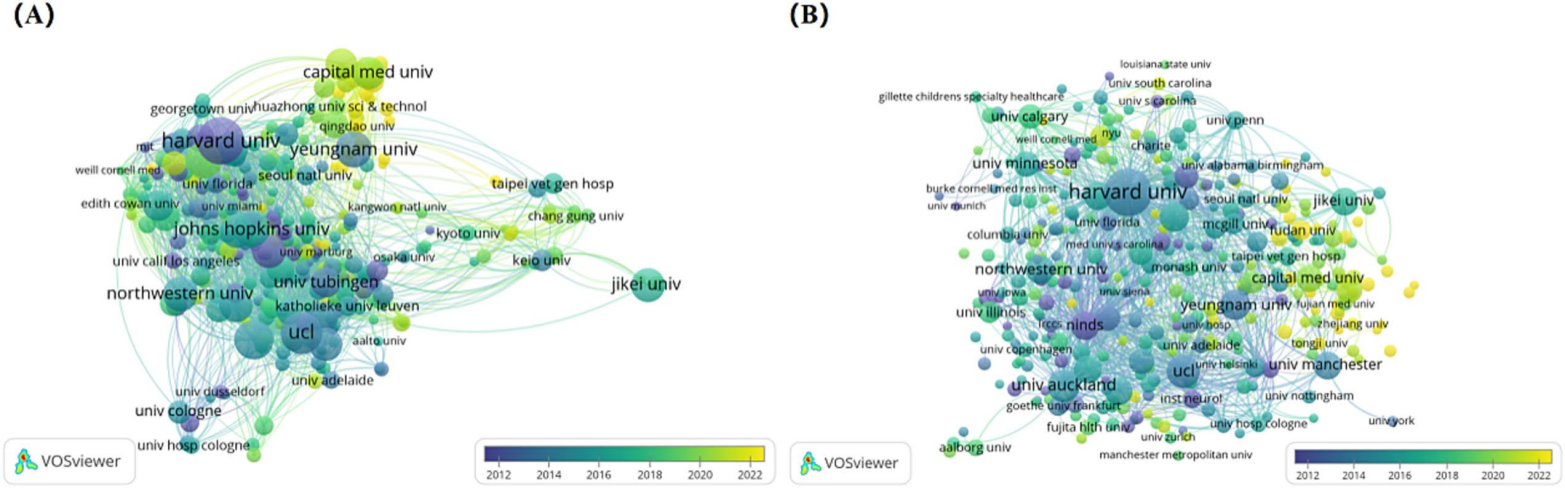
Analysis of institutions on NIBS for stroke. **(A)** Overlap visualization map of institutions co-authorship analysis generated by VOSviewer with the method linlog/modularity. Eight occurrences were included only. The purple and blue colors represented an early appearance, and the yellow color represented a late appearance. **(B)** Overlap visualization map of institutions’ citation analysis generated by VOSviewer with the method linlog/modularity. Eight occurrences were included only. The purple and blue colors represented an early appearance, and the yellow color represented a late appearance.

Figure 4A is the overlay visualization map of institutions co-authorship analysis and institutions’ citation analysis. Nodes represent institutions, while the color of the nodes represents the year of publication, referring to the color gradient in the bottom right corner of the figure. Harvard University, the National Institute of Neurological Disease and Stroke, and Johns Hopkins University are depicted in purple, indicating that these institutions are early researchers and have been more far-reaching research in the field. The yellow nodes likely represent emerging institutions in this area, with Fudan University noted as the institution with the highest number of publications in recent years. Meanwhile, it can be found that Harvard University has more collaborations with other institutions, primarily within the United States, which facilitates in-depth research in this field through close cooperation domestically. Figure 4B is the overlay visualization map of institutions’ citation analysis. Purple represents institutions with high citations in the early years, larger nodes correspond to greater influence. Yellow represents institutions with high citations in recent years, indicating that the research of these institutions may be more in line with the current trends and hotspots. Overall, the top three in terms of total link strength are Harvard University (9515), University College London (6856), and University of Auckland (5737), revealing the dominance of the United States and the United Kingdom in this academic field.

**Figure 5:**
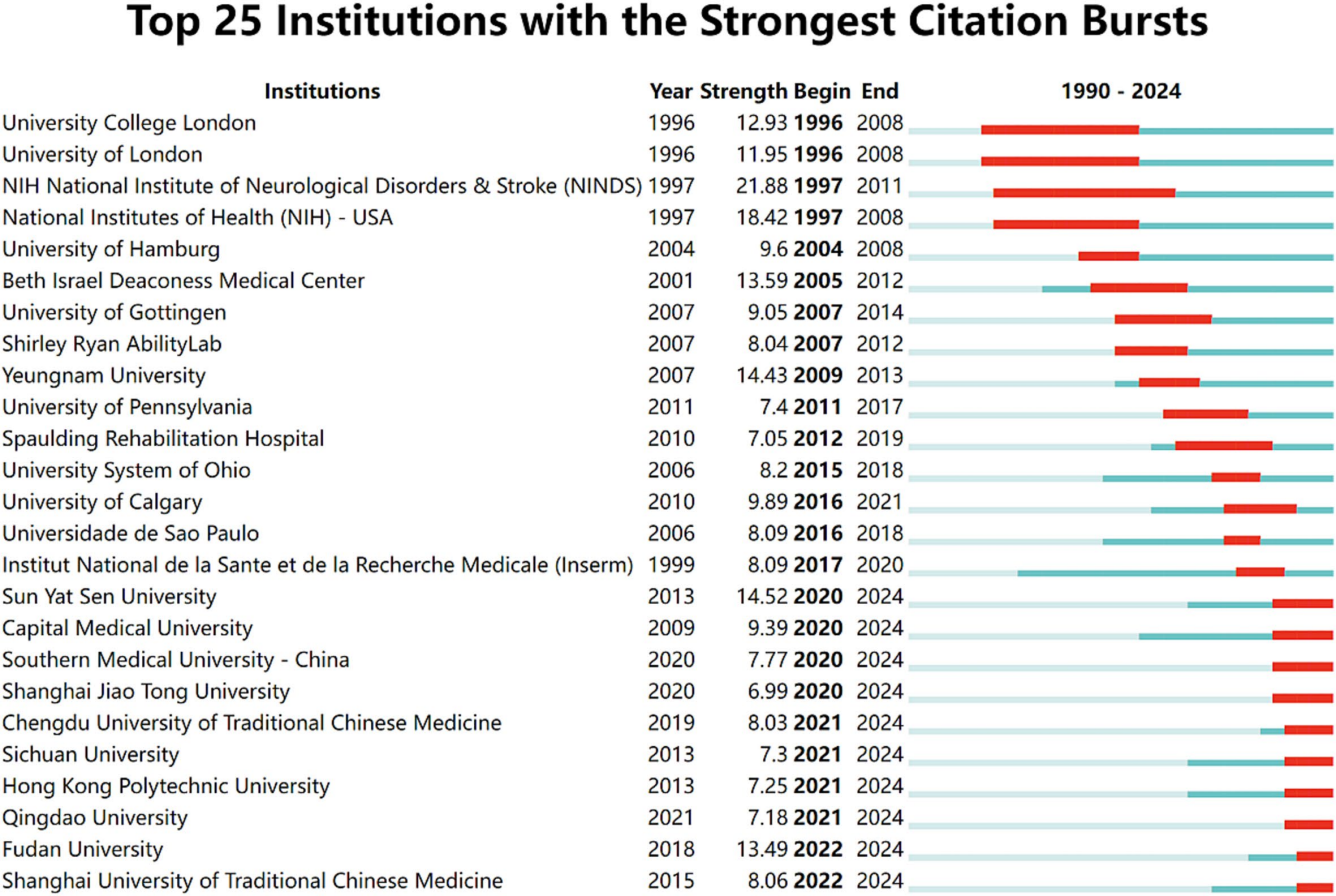
Top 25 institutions with the strongest citation bursts. Light blue indicates that the keyword has not yet appeared. Dark blue indicates that keywords have started to appear, but there have been no significant changes during the period. Red indicates the duration of the strongest citation.

In the institutional burst detection (Figure 5), the top three in terms of burst strength are NINDS (21.88), NIH (18.42), and Sun Yat-sen University (14.52), with the years of bursts primarily concentrated on 1997–2011, 1997–2008, and 2020–202, respectively. Notably, from 2020 to 2024, many Chinese institutions began to engage in research within this field, and it is expected that a large number of research outputs will be produced subsequently.

### 3.4 Analysis of authors

A total of 15,886 researchers participated in this study, contributing to 4,453 publications. Table 3 lists the top 10 authors in the field with 499 publications. Among these authors, Fregni F (Harvard Medical School), Abo M (Jikei University), and Jang SH (Yeungnam University) were the outstanding contributors with the highest number of publications. Notably, Fregni F and Pascual-Leone A were both from Harvard Medical School. Fregni F topped the list with 74 publications and 6,169 citations, while Pascual-Leone A ranked first in terms of H-index and second in total citations (5,604). The H-index is one of the metrics for evaluating the scholarly impact of a researcher, organization, or journal, which was considered a more comprehensive and fair measure of research impact (24). A higher H-index implies that the author’s articles have been extensively cited. It is no doubt that the academic achievements of the Harvard Medical School team in this field have been fully recognized.

**Table 3:** Top 10 authors with most publications regarding NIBS for stroke.

Rank	Authors	Institutions	Countries	Documents	Citations	Total link strength	H-index
1	Fregni F	Harvard Medical School	USA	74	6,196	126	99
2	Abo M	Jikei University	Japan	61	1,246	175	32
3	Jang SH	Yeungnam University	South Korea	61	1,065	72	47
4	Pascual-leone A	Harvard Medical School	USA	56	5,604	113	128
5	Byblow WD	University of Auckland	New Zealand	48	3,417	108	53
6	Cohen LG	National Institute of Neurological Disease and Stroke	USA	48	4,674	111	122
7	Stinear CM	University of Auckland	New Zealand	43	3,659	119	54
8	Kirton A	University of Calgary	Canada	40	1,181	84	30
9	Madhavan S	University of Illinois Chicago	USA	35	810	25	31
10	Kakuda W	International University of Health & Welfare	Japan	33	1,018	124	26

**Figure 6:**
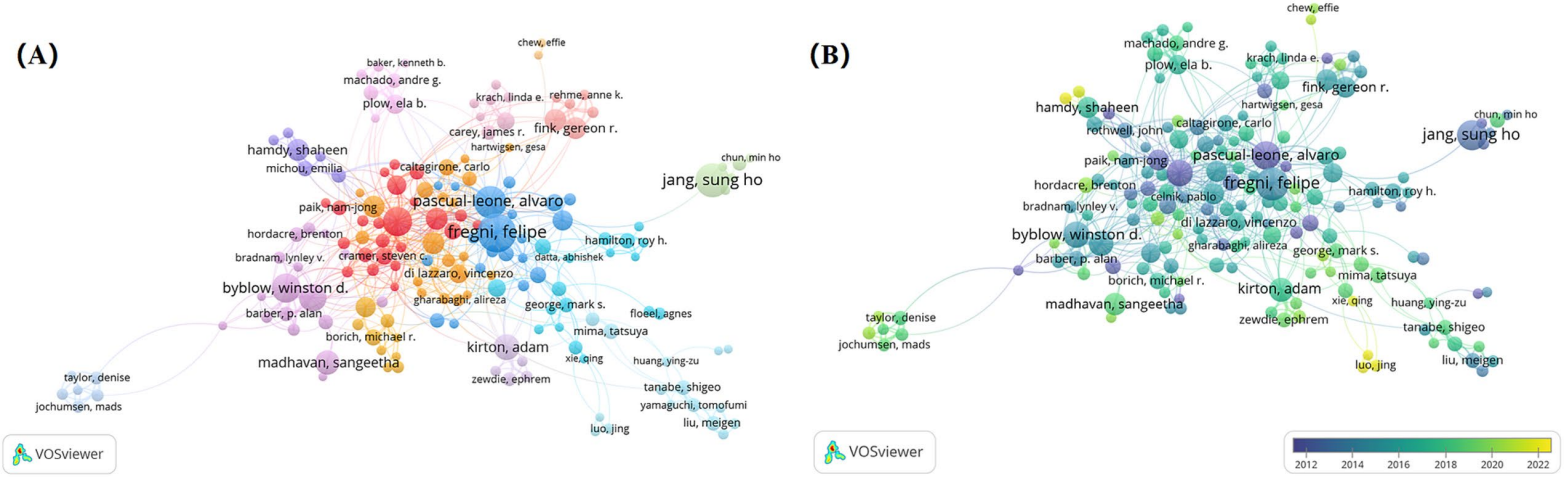
Analysis of authors on NIBS for stroke. **(A)** Network visualization map of authors’ collaboration analysis generated by VOSviewer with the method linlog/modularity. Eight occurrences were included only. **(B)** Overlap visualization map of authors’ collaboration analysis generated by VOSviewer with the method linlog/modularity. Eight occurrences were included only. The purple and blue colors represented an early appearance, and the yellow color represented a late appearance.

VOSviewer software was used to export network visualization and overlap visualization maps of authors’ collaboration. It reflects both the collaborative relationship between the authors and the timeliness of the research of the authors involved. The network visualization map of authors’ collaboration generated by VOSviewer (Figure 6A) indicated that NIBS for stroke rehabilitation has formed a range of core collaborations. Notably, Fregni F team (Harvard Medical School), Jang SH team (Jikei University), Cohen LG (University of Auckland), and Byblow WD (National Institute of Neurological Disease and Stroke) were four influential and relatively stable academic teams. The overlap visualization map of authors’ collaboration generated by VOSviewer (Figure 6B) shows that Luo J and Feng WW collaborated the most recently, with significant publications occurring between 2020 and 2022. It is reflected that this collaborative team may be a new star in the field, further indicating that the research of NIBS for stroke remains a prominent and important study hotspot.

**Figure 7:**
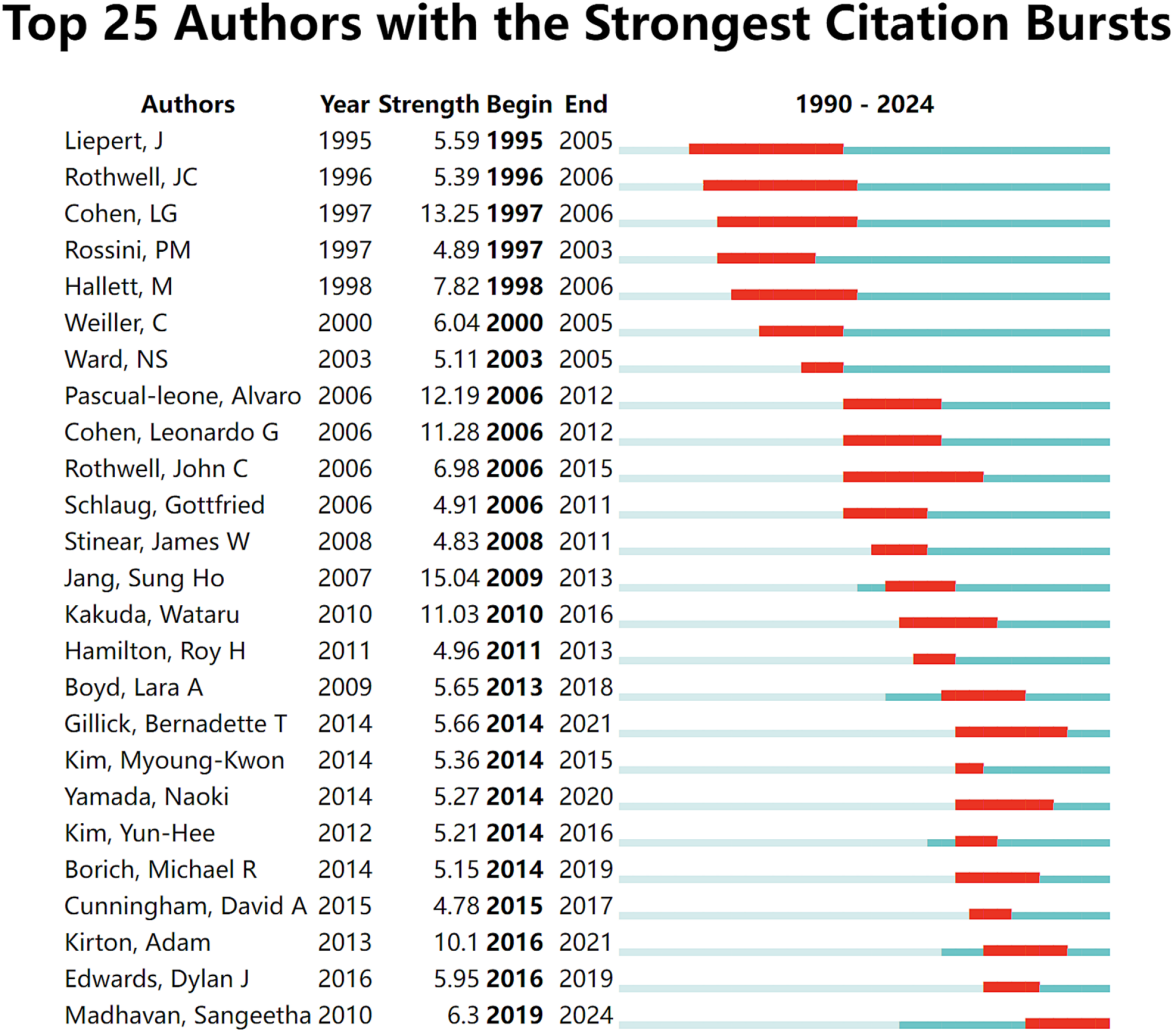
Top 25 authors with the strongest citation bursts. Light blue indicates that the keyword has not yet appeared. Dark blue indicates that keywords have started to appear, but there have been no significant changes during the period. Red indicates the duration of the strongest citation.

Figure 7 shows the top 25 authors with the strongest citation bursts. Jang SH, Cohen LG, and Pascual-leone A had the strongest bursts, indicating that they have created significant academic value for research related to NIBS in the field of stroke rehabilitation.

### 3.5 Analysis of journals

**Figure 8:**
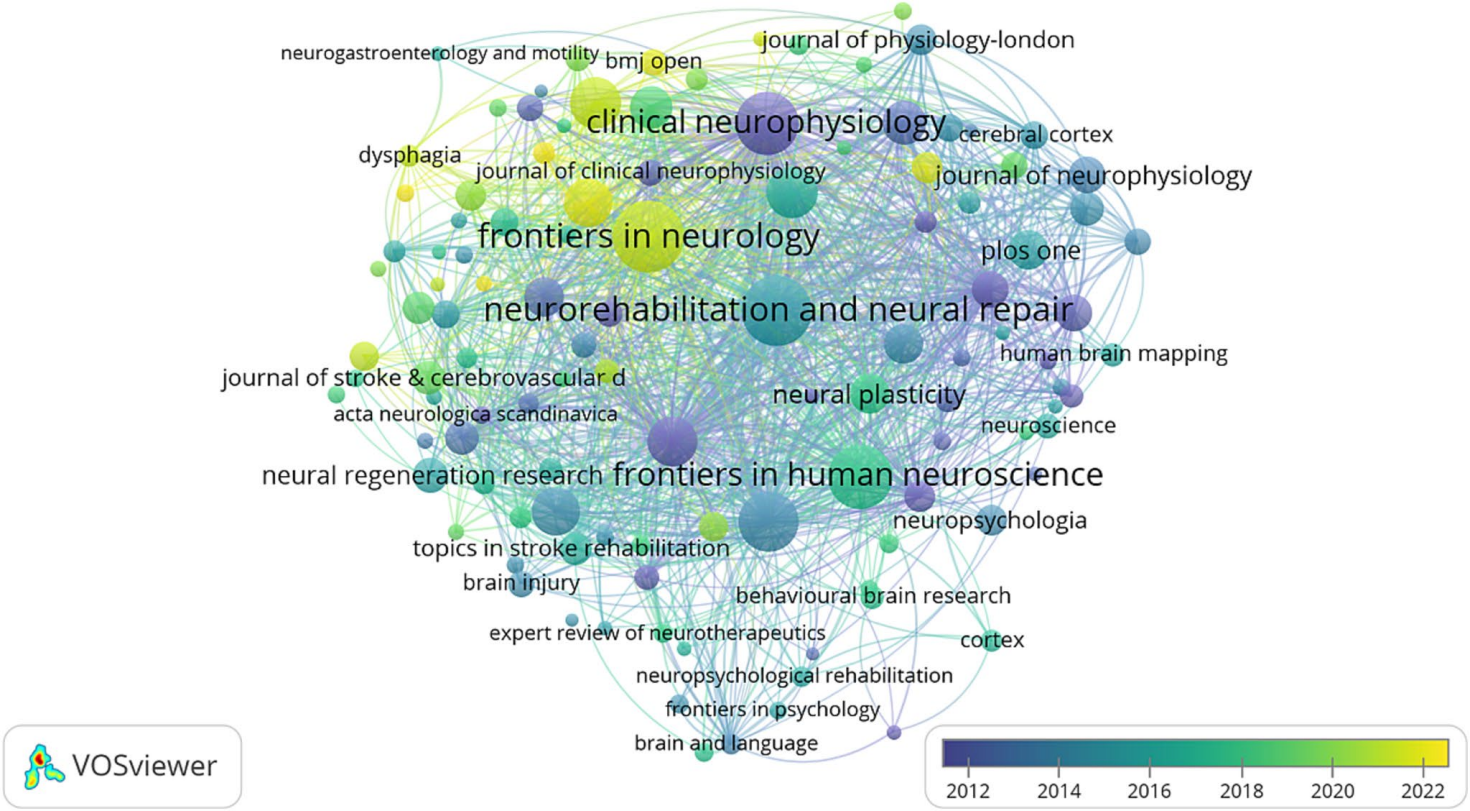
Overlap visualization map of journals analysis generated by VOSviewer with the method linlog/modularity. Eight occurrences were included only. The purple and blue colors represented an early appearance, and the yellow color represented a late appearance.

Journal publications are an important medium for the dissemination of scholarly knowledge and the exchange of learning, and this field of research has recently involved a total of 699 journals. Table 4 lists the top 10 journals with 1,111 articles on NIBS for stroke, accounting for 24.95% of the total literature. Among them, Frontiers in Neurology (160) had the highest number of publications, followed by Neurorehabilitation and Neural Repair (158), and Frontiers in Human Neuroscience (135). The top three in total citations were Clinical Neurophysiology (12,396), Stroke (10,896), and Neurorehabilitation and Neural Repair (8,074). In the Journal Citation Reports (JCR) partition, these journals accounted for 30% in Q1, 40% in Q2, 20%, and Q4 10%. The impact factor (IF) of the top ten journals in 2023 fluctuated from 1.9 to 7.8 points, with Stroke having the highest impact factor, as well as a significant number of total citations and Q1 partition, both of which confirmed the journal’s academic authority in the field. However, the overall impact factor remained low, with most journals below 4 points, which may be attributed to the relatively late emergence of modern rehabilitation, and related research is still in the slow development stage. Overall, the journals were closely linked to each other, with research covering a wide range of disciplines including neuroscience, physiology, and rehabilitation medicine. Frontiers in Neurology is probably the most popular journal in recent years (Figure 8).

**Table 4:** Top 10 journals with most publications regarding NIBS for stroke.

Rank	Journals	Countries	Documents	Citations	2023 impact factor	2023 JCR partition
1	Frontiers in neurology	Switzerland	160	1819	2.7	Q3
2	Neurorehabilitation and neural repair	USA	158	8,074	3.7	Q1
3	Frontiers in human neuroscience	Switzerland	135	4,499	2.4	Q2
4	Clinical neurophysiology	Ireland	130	12,396	3.7	Q2
5	Restorative neurology and neuroscience	Netherlands	116	4,562	1.9	Q4
6	Brain stimulation	USA	87	5,249	7.6	Q1
7	Frontiers in neuroscience	Switzerland	86	902	3.2	Q2
8	Stroke	USA	83	10,896	7.8	Q1
9	Brain sciences	Switzerland	79	484	2.7	Q3
10	Neurorehabilitation	Ireland	77	1884	1.7	Q2

### 3.6 Analysis of keywords

**Figure 9:**
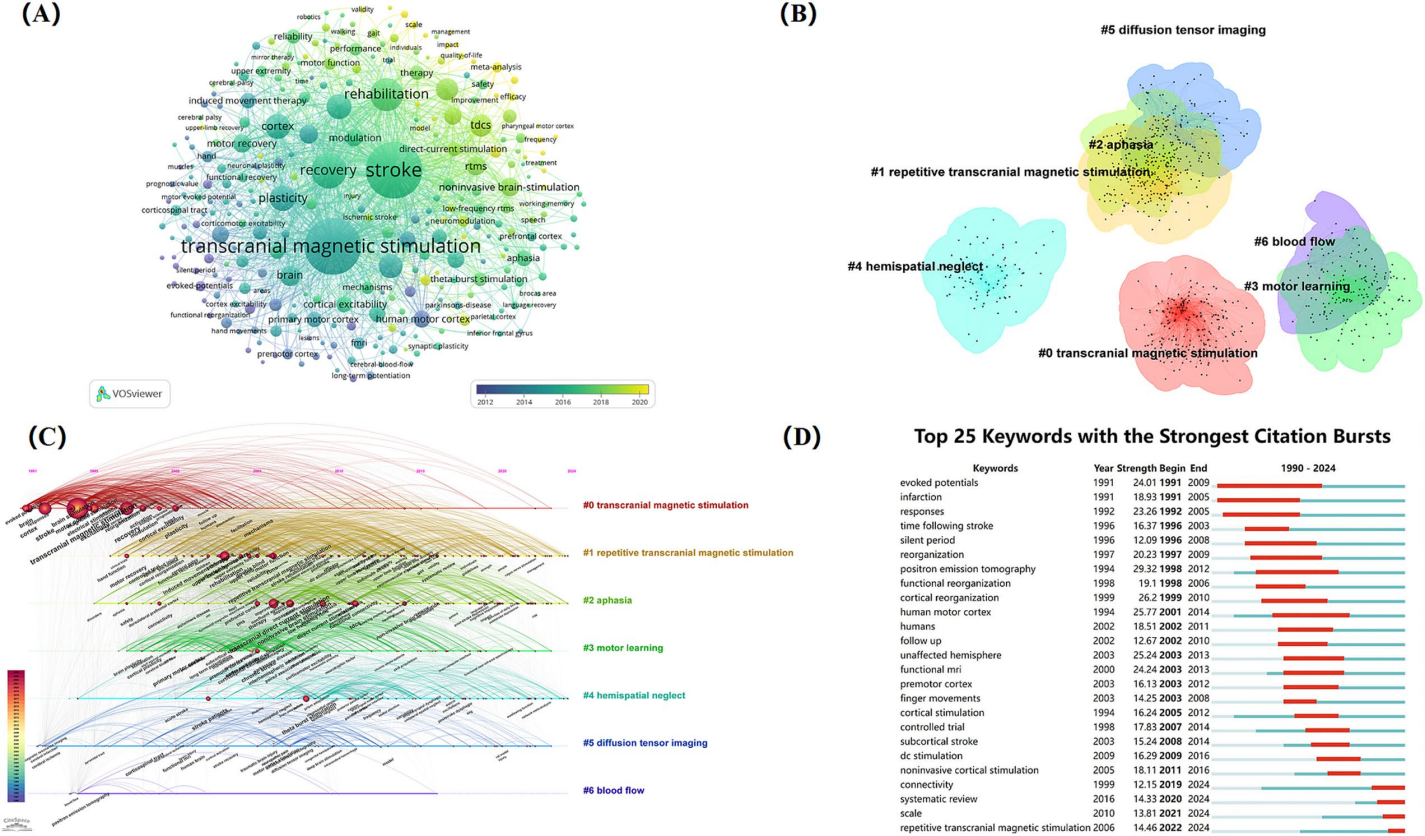
Analysis of keywords on NIBS for stroke. **(A)** Overlap visualization map of keywords co-occurrence analysis generated by VOSviewer with the method linlog/modularity. Thirty occurrences were included only. **(B)** Cluster map of keywords. **(C)** The timeline view of clustering analysis of keywords. **(D)** Top 25 keywords with the strongest citation bursts. Light blue indicates that the keyword has not yet appeared. Dark blue indicates that keywords have started to appear, but there have been no significant changes during the period. Red indicates the duration of the strongest citation.

The field of NIBS for stroke involved a total of 9,714 keywords, with 294 keywords appearing 30 times or more (Figure 9A). Among them, high-frequency keywords such as “stroke,” “transcranial magnetic stimulation,” “recovery,” “rehabilitation,” “plasticity,” and “excitability” highlighted their core position within the research. It can be seen that a series of new NIBS techniques for stroke rehabilitation have emerged in recent years, such as tDCS, rTMS, and theta-burst stimulation. Plasticity and excitability were the two major themes in mechanism research within this field, revealing the mechanisms of NIBS. Furthermore, aphasia and hemispatial neglect after stroke were more closely associated with TMS, providing a critical foundation for future in-depth research.

The cluster analysis of keywords is the categorization and summary analysis of the clusters of keyword terms with similar meanings in the literature, assisting researchers in quickly grasping the main research themes within the field (25). In this study, keywords related to NIBS for stroke were analyzed by using CiteSpace. A total of six keyword clustering labels were formed (Figure 9B): #0 transcranial magnetic stimulation, #1 repetitive transcranial magnetic stimulation, #2 aphasia, #3 motor learning, #4 hemispatial neglect, #5 diffusion tensor imaging, and #6 blood flow.

The timeline of clustering analysis is analyzed based on the cluster analysis of keywords, focusing on the historical span of the clustered keywords to provide researchers with insights into stage hotspots and development trends within this research field. The timeline view of clustering of keywords (Figure 9C) indicates that cluster #0 (transcranial magnetic stimulation) has the earliest appearance and the longest duration, signifying that transcranial magnetic stimulation has been the topic theme. After 1995, cluster #1 (repetitive transcranial magnetic stimulation) became active, suggesting that with the development of modern technology, more innovative NIBS techniques were further developed and applied in clinical practice. Cluster #2 (aphasia) and cluster #4 (hemispatial neglect) are also noteworthy, highlighting the exploration of the efficacy of NIBS for treating specific post-stroke dysfunction, which had some evidence-based value. Cluster #5 and cluster #6 have received comparatively less attention in recent years.

Figure 9D illustrates the top 25 keywords with the strongest citation bursts from 1991 to 2016. “Positron emission tomography” and “human motor cortex” have the highest burst strength of 29.32 and 25.77, respectively. Notably, “evoked potentials” was the earliest keyword with long duration and high burst strength. Additionally, the keywords “repetitive transcranial magnetic stimulation,” “connectivity, “and” scale “exploded during 2019–2024, suggesting that these may represent the research hotspot of NIBS in stroke rehabilitation in recent years.

### 3.7 Analysis of cited references

The analysis of reference co-citation can reflect the status and hotspots of research to some extent, and the highly cited references are regarded as groundbreaking foundations in this research field (26). Over the past 30 years, a total of 100,965 papers have been cited in this field, and Table 5 lists the 10 most frequently cited papers, which mainly explore important scientific issues like clinical applications, treatment modalities, and mechanisms of NIBS in stroke. These highly cited references were published between 1994 and 2014, with citation counts ranging from 310 to 579. “Influence of Interhemispheric Interactions on Motor Function in Chronic Stroke” stood out with 579 citations and the highest total link strength.

**Table 5:** Top 10 cited references with most publications regarding NIBS for stroke.

Rank	Reference title	First author (year)	Journals	Citations	Total link strength
1	Influence of interhemispheric interactions on motor function in chronic stroke	Nagako Murase (2004) (52)	Annals of Neurology	579	17,512
2	Excitability changes induced in the human motor cortex by weak transcranial direct current stimulation	M. A. Nitsche (2000) (36)	Journal of Physiology	558	14,137
3	Safety, ethical considerations, and application guidelines for the use of transcranial magnetic stimulation in clinical practice and research	Rossi et al. (102)	Clinical Neurophysiology	495	9,604
4	Effects of non-invasive cortical stimulation on skilled motor function in chronic stroke	Hummel et al. (103)	Brain	397	11,864
5	Non-invasive electrical and magnetic stimulation of the brain, spinal cord and roots: basic principles and procedures for routine clinical application	Rossini et al. (104)	Electroencephalography and Clinical Neurophysiology	382	6,136
6	Theta burst stimulation of the human motor cortex	Huang et al. (105)	Neuron	353	8,251
7	Sustained excitability elevations induced by transcranial DC motor cortex stimulation in humans	Nitsche and Paulus (106)	Neurology	327	9,270
8	Non-invasive brain stimulation: a new strategy to improve neurorehabilitation after stroke?	Friedhelm Hummel (2006) (54)	Lancet Neurology	316	9,122
9	Modulation of brain plasticity in stroke: a novel model for neurorehabilitation	Giovanni Di Pino (2014) (53)	Nature Reviews Neurology	313	7,680
10	Repetitive transcranial magnetic stimulation of contralesional primary motor cortex improves hand function after stroke	Takeuchi et al. (107)	Stroke	310	9,814

This was followed by “Excitability changes induced in the human motor cortex by weak transcranial direct current stimulation,” as well as “Safety, ethical considerations, and application guidelines for the use of transcranial magnetic stimulation in clinical practice and research.” The first authors of two papers were Friedhelm Hummel, with one of them, “Non-invasive brain stimulation: a new strategy to improve neurorehabilitation after stroke?” published in one of the top journals in the field. This paper argued that TMS and tDCS can be developed into valuable treatments in neurorehabilitation, but must be further evaluated in multicentral clinical trials.

**Figure 10:**
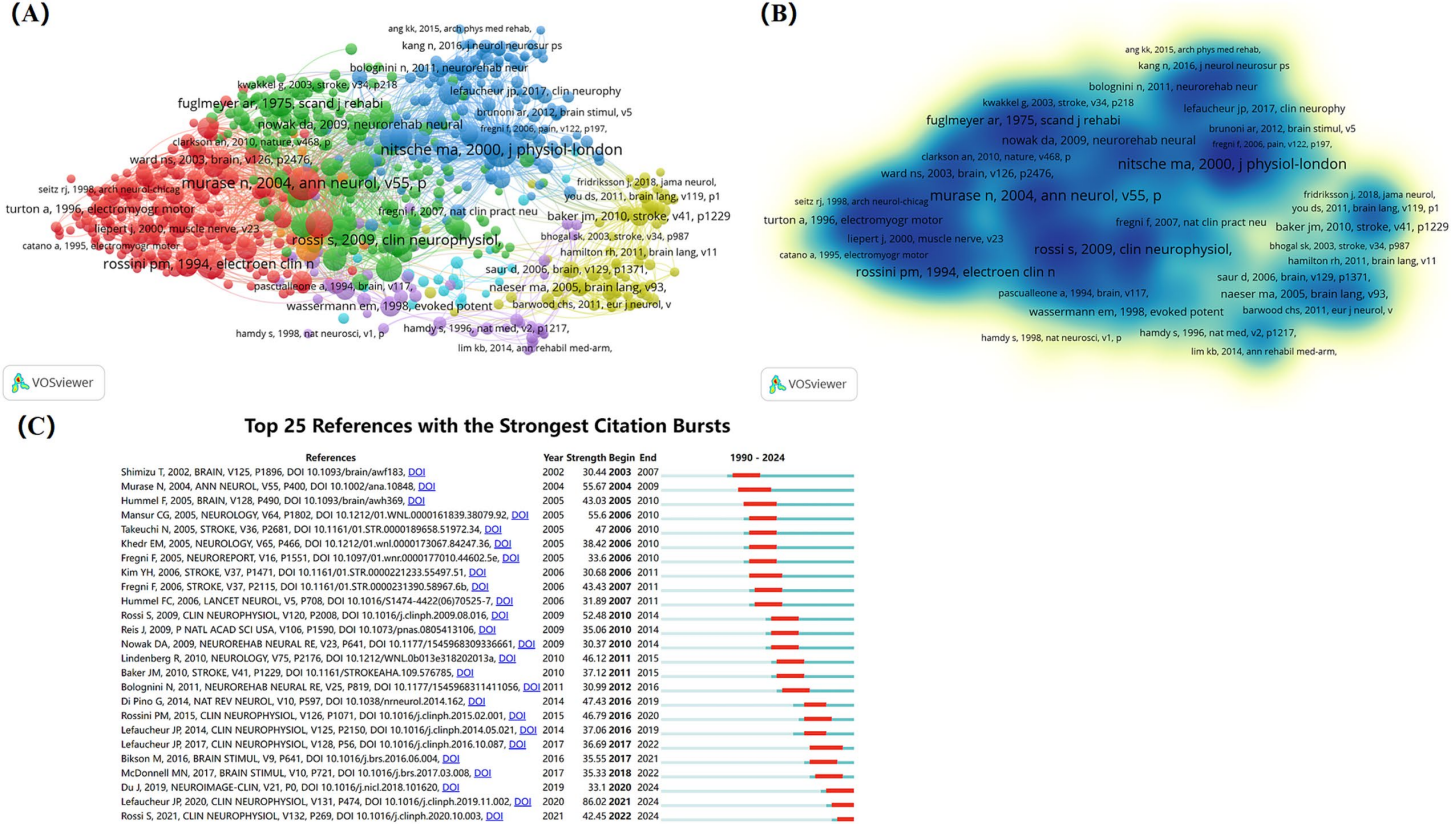
Analysis of cited references on NIBS for stroke. **(A)** Network visualization map of references co-citation analysis generated by VOSviewer with the method linlog/modularity. Forty occurrences were included only. **(B)** Density visualization map of references co-citation analysis generated by VOSviewer with the method linlog/modularity. Forty occurrences were included only. **(C)** Top 25 references with the strongest citation bursts. Light blue indicates that the keyword has not yet appeared. Dark blue indicates that keywords have started to appear, but there have been no significant changes during the period. Red indicates the duration of the strongest citation.

Network and density visualization map of references co-citation (Figures 10A,B) shows clustering into 7 categories with 666 nodes, 137,789 links, and 610,802 total link strength. Figure 10C shows the top 25 references with the strongest citation bursts, with the earliest citation burst occurring in 2002 and the most recent in 2022. Many references burst lasting about 4 or 5 years. The strongest burst was published in Clinical Neurophysiology, titled “Evidence-based guidelines on the therapeutic use of repetitive transcranial magnetic stimulation (rTMS): An update (2014–2018),” which introduces the latest guidelines for rTMS treatment, offering valuable insights for individualizing treatment and improving clinical efficacy.

### 3.8 Analysis of subject categories

**Figure 11:**
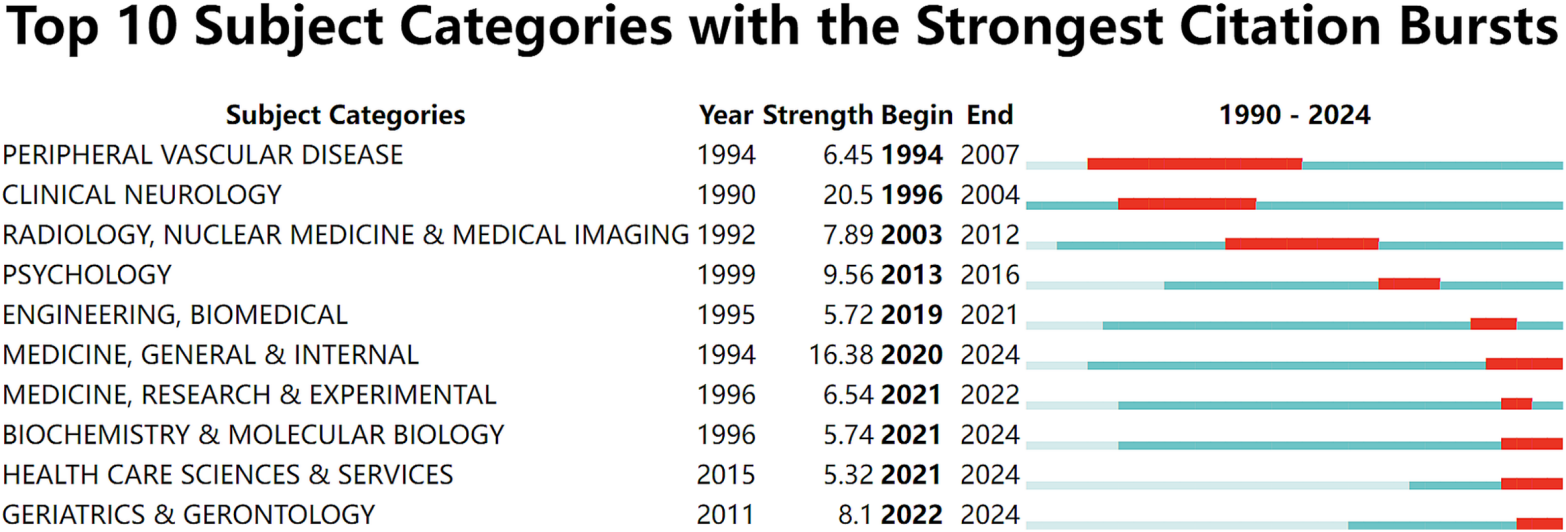
Top 25 subject categories with the strongest citation bursts. Light blue indicates that the keyword has not yet appeared. Dark blue indicates that keywords have started to appear, but there have been no significant changes during the period. Red indicates the duration of the strongest citation.

NIBS for stroke involved 121 subject categories, with its literature primarily concentrated in the fields of neurosciences, rehabilitation, psychology, and sport sciences. Table 6 lists the top 10 subject categories in this field, with neurosciences occupying the top one (244, 54.95%), followed by clinical neurology (1,579, 35.46%) and rehabilitation (17.22%). Figure 11 illustrates the top 25 subject categories with the strongest citation bursts. Notably, from 1994 to 2024, early research bursts in this field were predominantly concentrated in clinically oriented disciplines, such as peripheral vascular disease, clinical neurology, radiology, nuclear medicine & medical imaging. The research trend had a shift towards fundamental disciplines, including biochemistry & molecular biology, medicine, research & experimental, as well as social sciences like health care sciences & services, geriatrics & gerontology recently. It is suggested that the development of clinical, mechanistic, and social science research in NIBS for stroke is maturing with the progress of time.

**Table 6:** Top 10 subject categories with most publications regarding NIBS for stroke.

Rank	Subject categories of web of science	Record count	% of 4,453
1	Neurosciences	2,447	54.95
2	Clinical neurology	1,579	35.46
3	Rehabilitation	767	17.22
4	Psychology	213	4.78
5	Sport sciences	201	4.51
6	Medicine general internal	175	3.93
7	Peripheral vascular disease	166	3.73
8	Medicine research experimental	159	3.57
9	Physiology	147	3.30
10	Engineering biomedical	139	3.12

### 3.9 Analysis of funding agencies

A total of 3,068 organizations have provided funding for research in this area. Table 7 lists the top 10 organizations that provided the most funding for research in this field. The top three funding agencies were the United States Department of Health and Human Services (HHS), the National Institutes of Health (NIH), the National Natural Science Foundation of China (NSFC), with the United States being the major funding contributor to research in this field.

**Table 7:** Top 10 funding agencies with most publications regarding NIBS for stroke.

Rank	Funding agencies	Record count	% of 4,453
1	United States Department of Health and Human Services	581	13.05
2	National Institutes of Health	578	12.98
3	National Natural Science Foundation of China	258	5.79
4	Ministry of Education Culture Sports Science and Technology	154	3.46
5	Japan Society for The Promotion of Science	150	3.37
6	National Institute of Neurological Disorders Stroke	146	3.28
7	German Research Foundation	126	2.83
8	Grants in Aid for Scientific Research Kakenhi	113	2.54
9	UK Research Innovation	99	2.22
10	Canadian Institutes of Health Research	93	2.09

## 4 Discussion

### 4.1 Overview of results

This study analyzed 4,453 publications related to NIBS for stroke from 1985 to 2024, involving 82 countries/regions, 4,015 institutions, 15,886 researchers, and 699 journals. The publication trend can be broadly divided into three stages. From 1990 to 2010, the application of NIBS in stroke rehabilitation was in its infancy, and the related research was superficial. In the 1980s, Professor Anthony Barker of the University of Sheffield successfully applied non-invasive magnetic stimulation to elicit motor-evoked potentials, which opened the gate to a new world for research on transcranial magnetic stimulation in both fundamental and diagnostic fields (27). With the advancement of neuroimaging technology, researchers began to explore the higher brain functions of humans non-invasively (28). The most classic non-invasive brain stimulation technique, TMS, provided a new approach to scientific research by inducing “virtual lesions” (29). Arac’s team was the first to research TMS in stroke rehabilitation and explore its prognostic value (30). Although some studies have provided clinical evidence for the use of TMS in the treatment of stroke (31–34), the lack of standardization and convincing evidence regarding its effectiveness and the negative results from some studies have contributed to a relatively slow progression of research in this area (35). Fortunately, the interest of researchers remained high, probably due to the intuitive appeal of NIBS for their potential to promote neural plasticity (36). Between 2011 and 2018, the ever-evolving landscape of NIBS techniques led to an increase in clinical studies focused on stroke rehabilitation, broadening horizons for the development of the field. The number of publications in the field grew rapidly from 2019 to 2024, with a peak in 2022. This trend reflected a growing emphasis on NIBS for stroke rehabilitation by researchers, alongside the release of a series of expert consensus and guidelines (37, 38). Despite a slight decline in publications after 2022, the overall quantity is still considerable. It is not difficult to predict that future research in this area will continue its upward trajectory.

Currently, the United States, China, Germany, and the United Kingdom dominate this field, and the United States and Germany have the closest cooperation with other countries. Most of the active contributors were from developed countries, likely since NIBS techniques primarily originated in Europe and the United States. Moreover, developed countries have a high level of scientific research and abundant funding, providing important support for research in this field, which is an absolute advantage. Although China ranked second in the number of publications and has been increasingly active in more specific research in recent years, it lacked international cooperation, started late, and had less global influence. Surprisingly, half of the top 10 most productive institutions were from the United States, which may be attributed to the significant investment in fundamental research in the United States since 1950. The two major health agencies of the United States, HH and NIH, were key sources of funding for research in this area, further reaffirming the dominance and far-reaching influence of the United States. In recent years, China has strongly supported the development of public health and made rapid progress in this field, with Sun Yat-sen University as a typical representative. Over the past 10 years, Sun Yat-sen University has concentrated on NIBS for the rehabilitation of post-stroke dysphagia and aphasia, achieved a series of achievements and become a rising star in the academic landscape of this field (39–43).

Fregni F was a leader in terms of publications and citations, and his team’s main research interest was in exploring and developing NIBS techniques for the treatment of neurological disorders. Fregni F published a longitudinal cohort study protocol in 2021 that utilized assessment tools such as electroencephalography (EEG), functional near-infrared spectroscopy (fNIRS), TMS, and magnetic resonance imaging (MRI) to investigate biomarkers associated with dysfunction and to understand the impact LF-Rtms.

of rehabilitation on the neuroplasticity of the brain (44). Then with the help of EEG and TMS, the research further explored the relationship between the recovery of motor function of the upper limb and potential biomarkers, it is likely to provide significant value for accurate diagnosis, guidance of treatment, and disease prevention in stroke patients, making the study worthy of attention (45, 46). Fregni F and Pascual-leone A, both affiliated with Harvard University, have collaborated extensively and devoted to researching the efficacy and safety of the two classic NIBS techniques, tDCS and TMS, for stroke rehabilitation while exploring the optimal parameter or combination of each technique (47–49). In addition, Fregni F, as a leader in the field, has developed guidelines for the application of NIBS techniques in collaboration with researchers from Germany, Brazil, the United Kingdom, France, Italy, Denmark and other countries to assist clinicians in solving problems in clinical practice (50, 51). Broader international cooperation and high-quality outputs are expected in the future.

Of the top 10 highly productive journals, four were from Switzerland, three were in the United States, two were in Ireland, and one was in Netherlands, suggesting that these countries play a crucial role in advancing academic progress in the field. Three journals, Clinical Neurophysiology, Stroke, Neurorehabilitation and Neural Repair, ranked high in terms of total citations, all of which have a JCR partition of Q1 and primarily focus on clinical neurophysiology and neurorehabilitation research related to NIBS for the treatment of stroke, which are influential and noteworthy by researchers. In general, the impact factors of the journals typically ranged from 2 to 4 points, indicating that publishing high-quality studies in influential journals is still a challenge.

In the subject category, articles related to NIBS for stroke were mainly on neuroscience, clinical neurology and rehabilitation, which once again emphasized that stroke is a vascular disease that seriously affects human health and requires long-term neurorehabilitation management to improve dysfunction for alleviate the heavy burden. Among the top ten cited references, the one with the highest citation and total link strength was “Influence of Interhemispheric Interactions on Motor Function in Chronic Stroke” by Cohen LG, which describes the transcallosal effect in the lesioned hemisphere of patients with chronic stroke, laying the foundation for subsequent mechanistic research (52). It is worth noting that two articles published in Lancet and Nature (53, 54) found that NIBS can be used to monitor and regulate the excitability of neuronal circuits in the cortex, which partially validates the efficacy of NIBS in post-stroke rehabilitation. In recent years, the two publications with the strongest citation bursts are the expert guidelines on TMS and rTMS (37, 55). Meanwhile, we observed that a clinical practice guideline on stroke rehabilitation management, published in the Annals of Internal Medicine at the beginning of 2025, was cited 239 times within just 2 months. This indicates high attention among researchers in the standardized application of NIBS (56). But owing to the variability and complexity of the brain in different individuals, the efficacy of NIBS made a difference, which was a tough problem. Researchers should take individualized treatment protocols of NIBS for patients seriously.

### 4.2 Research hotspot analysis

Through co-occurrence, clustering, and burst analysis of keywords, it was found that the research hotspots and emerging trends in the field of NIBS for stroke were mainly concentrated in three aspects: the types, clinical indications, and assessment techniques of NIBS.

Currently, the mainstream technologies of NIBS mainly include two categories: TMS and Transcranial Electrical Stimulation (TES). TMS is primarily categorized into sTMS, pTMS, rTMS; TES is mainly classified into four categories: tDCS, transcranial alternating current stimulation (tACS), transcranial pulsed current stimulation (tPCS) and transcranial random noise stimulation (tRNS). In 2016, the American Heart Association/American Stroke Association issued specialized guidelines for stroke rehabilitation, recommending TMS and tDCS based on moderate-quality clinical trial evidence. This guideline emphasizes the adjunctive role of NIBS in the rehabilitation of chronic stroke. However, due to a lack of safety data and high-quality evidence, the use of NIBS was not recommended during the acute phase (57).

Clusters #0 and #1 constitute the core axis of the technical methodology. TMS serves as a fundamental tool for neuromodulation, concentrating its research on the mechanisms of cortical excitability regulation, whereas rTMS is primarily concerned with the optimization of therapeutic parameters. It is worth noting that rTMS was the most popular non-invasive brain stimulation technique in recent years. It may be attributed to the fact that rTMS can deliver repetitive, continuous and regular stimulation to effectively excite more horizontally oriented neurons. A recent meta-analysis demonstrated that rTMS significantly alleviated various dysfunctions after stroke and improved the quality of life for patients, and suggested that clinicians should incorporate it into rehabilitation protocol for post-stroke individuals (58). Some scholars have concentrated on the stimulation modes of rTMS. A qualitative systematic review indicated that rTMS applied to the injured hemisphere can ameliorate post-stroke motor deficits, but its parameters remain uncertain (59). Several clinical studies have found that both high-frequency repetitive transcranial magnetic stimulation (HF-rTMS) and low-frequency rTMS (LF-rTMS) can modulate the excitability of the cerebral motor cortex, thereby improving the motor function in the early stage of stroke, and HF-rTMS was more effective than LF- rTMS (60–62). In post-stroke aphasia, the LF-rTMS had a more immediate effect (63, 64). Thus, it is evident that the selection of rTMS modes is closely associated with the site of injury, the type of disorder and the stage of the disease. Furthermore, there is an urgent need for large-scale, high-quality and long-term studies to explore the optimal parameters of rTMS in stroke treatment. A Canadian multidisciplinary team established the CanStim platform and formulated expert consensus on rTMS for upper limb motor dysfunction after stroke. They proposed a standardized framework for clinical trials that emphasizes precise targeting, strict blinding, and multi-dimensional outcome assessments. This work has also laid the foundation for the standardized and normative clinical application and evidence-based transformation of rTMS (65).

While the development of TES has lagged behind that of rTMS, the studies of tDCS for stroke continue to rise. Compared to rTMS, tDCS is characterized by its cost-effectiveness, convenience, and ease of administration, which positions it as a promising adjunctive therapy for stroke rehabilitation. Guidelines have been established to provide technical implementation protocols for the remote application of tDCS in clinical research (66). tDCS may provide certain benefits in improving post-stroke dysfunction, however, the current evidence is insufficient to clearly delineate its therapeutic potential (19). Furthermore, the efficacy of tDCS may be influenced by various stimulation parameters, and the complex interactions among these parameters could result in inconsistent outcomes in post-stroke patients, a phenomenon akin to that observed with rTMS (67). Therefore, recent research hotspots have focused on the exploration of parameter standardization. A systematic review of parallel randomized clinical trials indicates that anodal transcranial direct current stimulation (a-tDCS) is a relatively safe intervention for patients with ischemic stroke, facilitating the recovery of upper limb function in these individuals (68). Another study has confirmed this finding and revealed that a-tDCS can improve cerebral hemodynamics in patients with cerebral ischemia (69). Furthermore, the combined application of two different NIBS techniques can enhance clinical efficacy. Preliminary findings suggested that the rTMS-tDCS may be more effective than rTMS alone (70, 71). The latest expert consensus suggests that, despite the increasing clinical practices and treatment guidelines indicating the potential therapeutic benefits of NIBS in improving post-stroke dysfunction, there are evidence-based translation barriers that limit their progress in the rehabilitation field. Simultaneously, this consensus provides a systematic framework for the application of TMS and tDCS in stroke rehabilitation, thereby accelerating the transition from laboratory to clinical practices and ultimately achieving precision and universalization in stroke rehabilitation (38).

From the clustering analysis of keywords, patients with post-stroke aphasia and hemispatial neglect were major subjects of NIBS. Post-stroke aphasia typically affects the language area of the dominant hemisphere. Commonly low-frequency rTMS in Broca’s area of the non-dominant hemisphere and tDCS of the bilateral hemispheres are applied to the treatment of post-stroke aphasia (72). Two meta-analyses reported consistently that the combination of NIBS techniques with speech training effectively enhances the speech function of patients with post-stroke aphasia (73, 74). Given that the recovery of the language network is dynamic and influenced by various factors, the underlying mechanisms of NIBS in post-stroke aphasia remain an unresolved issue. Shah P (75) proposed that a more comprehensive understanding of the potential of NIBS for post-stroke aphasia should integrate neuroimaging and electrophysiological measures.

Hemispatial neglect is a common cognitive dysfunction after stroke. Generally, the location of TMS and tDCS was mostly in the posterior parietal cortex, specifically the P3 and P4 regions of the EEG 10–20 localization system. The stimulation modes can be broadly categorized into two types: single stimulation of either the unaffected or affected cerebral hemisphere and simultaneous stimulation of both cerebral hemispheres. Some researchers have combined NIBS with feedback training or robot-assisted therapy to treat hemispatial neglect and found that it can effectively improve the relevant symptoms of patients (76–78). However, compared with post-stroke aphasia, there were relatively few studies on post-stroke hemispatial neglect, suggesting that hemispatial neglect may have the potential to become a major direction in the future.

Motor learning depends on a time-sensitive window of synaptic plasticity. NIBS directly influences the neural circuits involved in motor learning by modulating neural plasticity in specific brain regions, which holds significant value in rehabilitation, sports training, and neuroscience research. In patients of chronic stroke, the synergistic effect of NIBS combined with task-oriented training can effectively enhance clinical outcomes (79, 80). Clusters #5 and #6 indicated that current research highlights the predictive value of neuroimaging in assessing treatment response. Prior studies have utilized Arterial Spin Labeling (ASL) and Diffusion Tensor Imaging (DTI) to identify alterations in global cerebral blood flow (CBF) and fractional anisotropy (FA), investigating the effects of rTMS on motor impairment in stroke patients. The findings suggest that significant regions associated with the reorganization of motor function in the brains of stroke patients exhibit changes following rTMS intervention (81). In recent years, the development of neurophysiology and neuroimaging has become more mature. Many studies have combined NIBS with fMRI, EEG, fNIRS. It can not only monitor the neuronal activity of patients in real-time, enhance the accuracy of brain function assessment and provide precise treatment for stroke patients, but also further explore the mechanism of NIBS.

### 4.3 Future research trends

Future research trends in NIBS for stroke rehabilitation primarily focus on three aspects: deepening the understanding of underlying mechanisms, enhancing clinical efficacy through multimodal combined interventions, and overcoming technical challenges through multidisciplinary collaboration.

The therapeutic mechanisms of NIBS are complex and not yet fully understood, while cortical excitability and synaptic plasticity are two popular themes in NIBS mechanism research. Based on the stroke model of interactive interhemispheric inhibition, an appropriate increase in excitability of the affected side, coupled with inhibition of the healthy side, contributes to the functional recovery of the affected hemisphere (82, 83). NIBS can achieve a new balance in cortical excitability between the cerebral hemispheres and promote functional recovery after stroke (83, 84). Stroke is closely related to synaptic plasticity. Several studies have found that NIBS can induce long-term potentiation (LTP) and upregulate neuroplasticity-related proteins, such as brain-derived neurotrophic factor (BDNF) or N-methyl-d-aspartate receptor (NMDAR), which enhance plasticity of hippocampal protrusion and facilitate neurorepair (85–87). With the introduction of the brain network concept and the increasing number of related studies, researchers are beginning to recognize that stimulation generated by NIBS may exert therapeutic effects by affecting the functional (88) connectivity of brain networks (89, 90). This explains why the keyword “connectivity” was exploding from 2019 to 2024, and further exploration may bring surprising results. The ongoing development of brain networks in the field of NIBS is expected to inspire new research directions and bring promising prospects for stroke patients.

Multimodal combined interventions are poised to become a hotspot in future research. The essence of rehabilitation for neurological deficits after stroke lies in reactivating neuroplasticity and reconstructing functional networks. However, traditional single therapies often fall short of achieving comprehensive recovery due to their limited target effects and insufficient synergy. The introduction of multimodal combined interventions effectively solves the limitations of single therapies by integrating NIBS with synergistic approaches such as exercise training, virtual reality (VR), biofeedback, and acupuncture. For example, the multisensory input from VR can stimulate the cooperative functioning of multiple brain regions, including the default mode network and the dorsal attention network, while NIBS enhances task-related neural synchrony through targeted stimulation of key nodes, thereby improving motor dysfunction after stroke (91). As a method of complementary and alternative medicine, acupuncture has gained widespread acceptance in stroke rehabilitation in recent years (92). A meta-analysis revealed that the combination of acupuncture and rTMS significantly enhances motor function and daily living abilities in stroke patients (93). Although NIBS techniques are increasingly utilized in stroke rehabilitation research, their clinical effectiveness varies significantly due to several factors, including stimulation parameters, brain anatomy, lesion location, severity, genetics, and so on (94). The quantification of these factors can be enhanced through the integration of neuroimaging techniques. For example, the combination of EEG and fNIRS can provide a more accurate representation of the functional activities of brain neural networks, and new software developed from magnetic resonance technology can optimize the stimulation parameters of tDCS (95–97). The multimodal combined intervention of NIBS not only offers valuable insights into the mechanisms of stroke pathophysiology and the recovery of neurological function but also facilitates the possibility of personalized precision rehabilitation.

However, the clinical translation of multimodal combined interventions continues to face challenges. Firstly, the combined use of NIBS and neuroimaging instruments may present several issues, including magnetic field interference, radio frequency noise, current leakage, interference of patient movement, and so on (98). Secondly, the high cost of NIBS equipment and its dependence on professional operation limit its widespread adoption in primary healthcare. Therefore, interdisciplinary collaboration across neurology, rehabilitation, engineering, sociology, and geriatrics will be crucial for the development of NIBS in stroke rehabilitation, which aligns with the results of the disciplinary category analysis. Currently, researchers are continuously enhancing the development and design of technologies such as TMS-EEG, TMS-fMRI, and TMS-EEG-fMRI (99–101). However, this process still requires significant time and multidisciplinary knowledge. Furthermore, researchers strongly advocate for increased collaboration among scientists from diverse disciplinary backgrounds to provide scientific evidence supporting the efficacy of NIBS in improving clinical effects for stroke patients and reducing the burden on healthcare systems.

### 4.4 Limitations

This study has several limitations. Firstly, the search was confined to the WoSCC database. While the WoSCC database for high-quality bibliometric analyses is widely accepted among researchers, there are still some potentially relevant studies that were not included, which may have led to some selection bias. Secondly, the type of language was restricted to English, which may have resulted in a lack of comprehensiveness. Lastly, high-quality literature often requires time to achieve the expected citations, meaning that some recent high-quality studies may not yet have received sufficient attention. These limitations should be taken into account when interpreting the results of this study. It is anticipated that better analysis with updated and intact data will be conducted in the future to understand comprehensively the research dynamics in this field.

## 5 Conclusion

This study analyzed the relevant literature on NIBS for stroke from 1985 to 2024 by searching the WoSCC database and using bibliometric tools, which helps researchers understand the research dynamics in this field. NIBS technology holds great potential in stroke rehabilitation and has a wide landscape of development. Moving forward, it is essential to strengthen international cooperation between countries and conduct more high-quality and high-level studies, which will help the further advancement of this field.

## Data Availability

The original contributions presented in the study are included in the article/Supplementary material, further inquiries can be directed to the corresponding author.

## Footnotes

^1^ https://www.ncbi.nlm.nih.gov/mesh/

^2^ https://charticulator.com/
