# Supplementary material for: Emerging trends and research hotspots of non-invasive brain stimulation for stroke: a bibliometric and visualization study
Source: Front Neurol. 2025 May 20;16:1540405. doi: 10.3389/fneur.2025.1540405 (PMC12132806; doi:10.3389/fneur.2025.1540405)
Supplement: Supplementary file 1 [file Table_1.docx]

***Supplementary Material***

**Supplement 1.** Search strategy

| **Search** | **Query** | **Items** |
| --- | --- | --- |
| #1 | Search: TS=(“Stroke*” OR “Cerebral Hemorrhag*” OR “Brain Infarct*” OR “Brain Ischemia” OR “Cerebral Infarct*” OR “Cerebrovascular Accident*” OR “CVA” OR “Apoplexy” OR “Cerebrovascular Apoplexy” OR “Apoplexy, Cerebrovascular” OR “Vascular Accident*, Brain” OR “Brain Vascular Accident*” OR “Cerebrovascular Stroke*” OR “Stroke*, Cerebrovascular” OR “Cerebral Stroke*” OR “Stroke*, Cerebral” OR “Stroke*, Acute” OR “Acute Stroke*” OR “Acute Cerebrovascular Accident*” OR “Cerebrovascular Accident*, Acute” OR “Hemorrhag* Stroke*” OR “Stroke*, Hemorrhag*” OR “Intracerebral Hemorrhag* Stroke*” OR “Hemorrhag* Stroke*, Intracerebral” OR “Stroke*, Intracerebral Hemorrhag*” OR “Isch?emic Stroke*” OR “Stroke*, Isch?emic” OR “Cryptogenic Ischemic Stroke*” OR “Ischemic Stroke*, Cryptogenic” OR “Stroke*, Cryptogenic Ischemic” OR “Cryptogenic Stroke*” OR “Cryptogenic Embolism Stroke*” OR “Embolism Stroke*, Cryptogenic” OR “Stroke*, Cryptogenic Embolism” OR “Wake-up Stroke*” OR “Stroke*, Wake-up” OR “Wake up Stroke*” OR “Acute Ischemic Stroke*” OR “Ischemic Stroke*, Acute” OR “Stroke*, Acute Ischemic” OR “Embolic Stroke*” OR “Stroke*, Embolic” OR “Stroke*, Thrombotic” OR “Thrombotic Stroke*” OR “Acute Thrombotic Stroke*” OR “Stroke*, Acute Thrombotic” OR “Thrombotic Stroke*, Acute” OR “Infarct*, Brain”) | 509,386 |
| #2 | Search:TS=(“Magnetic Stimulation*,Transcranial” OR “Stimulation*, Transcranial Magnetic” OR “Transcranial Magnetic Stimulation*” OR “Transcranial Magnetic Stimulation*, Paired Pulse” OR “Transcranial Magnetic Stimulation*, Repetitive” OR “Transcranial Magnetic Stimulation*, Single Pulse” OR “*TMS” OR “Anodal Stimulation Transcranial Direct Current Stimulation” OR “Anodal Stimulation tDCS*” OR “Stimulation tDCS*, Anodal” OR “tDCS*, Anodal Stimulation” OR “Cathodal Stimulation Transcranial Direct Current Stimulation” OR “Cathodal Stimulation tDCS*” OR “tDCS” OR “Stimulation tDCS*, Cathodal” OR “tDCS*, Cathodal Stimulation” OR “Transcranial Alternating Current Stimulation” OR “Transcranial Random Noise Stimulation” OR “Repetitive Transcranial Electrical Stimulation” OR “Transcranial Electrical Stimulation” OR “Electrical Stimulation*, Transcranial” OR “Stimulation*, Transcranial Electrical” OR “Transcranial Electrical Stimulation*” OR “theta burst stimulation” OR “non-invasive brain stimulation” OR “galvanic vestibular stimulation” OR “coordinated reset vibroactile stimulation”) | 83,238 |
| #3 | #1 AND #2 | 5,030 |
